# Intrauterine death from placental abruption: influence of labor duration and mode of delivery on maternal outcomes

**DOI:** 10.3389/fmed.2025.1530062

**Published:** 2025-01-22

**Authors:** Louis Van Hees, Robin Danger, Caroline Turbelin

**Affiliations:** ^1^Service de Gynécologie-Obstétrique, CHU de Fort de France, Fort-de-France, France; ^2^Service de Gynécologie-Obstétrique, CHA Libramont, Libramont-Chevigny, Belgium; ^3^Service d’anestéshie-réanimation, CHU de Fort de France, Fort-de-France, France; ^4^Service d’anestéshie-réanimation CHU Angers, Angers, France

**Keywords:** placental abruption, cesarean delivery, intrauterine fetal death, post-partum hemorrhage, vaginal delivery, complication

## Abstract

**Introduction:**

Intrauterine fetal death (IUFD) caused by retroplacental hematoma (RPH) can lead to serious maternal complications including coagulopathies and hemorrhage. A prolonged diagnostic-to-expulsion delay may increase coagulation risks. Vaginal delivery (VD) prolongs this delay, while cesarean delivery poses higher hemorrhagic risks. This study aimed to assess the relationship between expulsion delay and complications, identify a time threshold for increased risks, and evaluate VD feasibility.

**Material and methods:**

We conducted a retrospective single-center study at the University Hospital of Martinique, including all patients presenting with RPH and IUFD between January 2003 and December 2022. Complications were defined by a composite criterion, including the need for blood transfusion, severe anemia or admission to an intensive care unit.

**Results:**

VD was attempted in 26 of the 27 patients included and successfully achieved in 85% of the cases. All cesarean deliveries resulted in complications (*p* = 0.12), with 50% experiencing severe hemorrhage, compared to 21.7% of VD cases (*p* = 0.23). No statistically significant association was found between the expulsion delay and the occurrence of complications (*p* = 0.20). A low fibrinogen level upon admission was associated with an increased risk of severe hemorrhage (*p* = 0.01) and complications (*p* = 0.01).

**Conclusion:**

In this study, no evidence was found to suggest an association between the diagnostic-to-expulsion interval and maternal complications. Low fibrinogen levels at admission appear to be a prognostic factor for severe hemorrhage and could have therapeutic implications. Vaginal delivery remains feasible even in the presence of coagulopathies at admission.

## 1 Introduction

Retroplacental hematoma (RPH) predominantly occurs in the second half of pregnancy and refers to the premature detachment of a normally implanted placenta. In industrialized countries, the incidence of RPH ranges from 0.4 to 2% of births, while it can reach up to 6% in developing countries (1–3). According to the medical literature, the rate of intrauterine fetal death (IUFD) associated with RPH varies between 4.5 and 52% (4, 5).

The benefit of prompt fetal extraction on maternal and fetal outcomes in cases of RPH is well established (6). However, there is no clear consensus regarding the anesthetic and obstetric management of patients when RPH is associated with IUFD, despite the increased risk for maternal coagulopathies and complications in this situation (7).

The activation of coagulation and the fibrinolytic system during placental abruption is primarily due to the introduction of tissue factors into the maternal circulation, originating from the trophoblast, amniotic fluid, and decidua (8, 9). Coagulopathies are present in 20–30% of RPH cases, and their severity is proportional to the extent of placental detachment (6).

Hypofibrinogenemia is correlated with the severity of RPH as it reflects the extent of fibrin deposition between the uterus and placenta and seems to be a good indicator for early transfusion of blood products (10, 11). Furthermore, hyperfibrinolysis leads to the production of fibrin degradation products, which promote uterine atony and exacerbate bleeding (9).

It seems necessary to remove the site of tissue factor production to limit the worsening of hemostatic disorders. A delay of 6–8 h for expulsion is sometimes mentioned in the literature, but this recommendation is not universally supported by other studies (6).

The management of patients with IUFD complicating an RPH is poorly described in the literature, and it remains uncertain whether cesarean delivery (CD) improves the prognosis. The 2024 French guidelines for the management of IUFD indicate that special attention should be given to these patients, with labor initiation without delay. However, no specific recommendations have been made regarding the exact method of labor induction, labor management, or indications for CD (12).

Previous abruptio placentae, maternal hypertensive disease, substance abuse and cigarette smoking are well known risk factors (13). The prevalence of preeclampsia in Martinique (3.4%) during the period from 2010 to 2019 is significantly higher than that in mainland France (2%) (14). Additionally, as Martinique serves as a hub for cocaine trafficking between South America and Europe, the island faces substantial drug use, particularly in the form of crack cocaine. The convergence of these factors has shaped the expertise of our team.

The primary objective of this study is to analyze the delivery delay and determine a potential time threshold at which complications may be more prevalent. This will therefore challenge the current recommendation of 6–8 h.

Secondly, our goal is to provide evidence supporting the feasibility of VD even in cases of coagulopathy upon admission. In this context, the study compares the morbidity associated with VD and CD.

## 2 Methods

This is a retrospective, monocentric observational study, collecting data from the medical records of patients treated at the University Hospital Center of Martinique, Fort-de-France, from January 1, 2003, to December 31, 2022.

Patient records with the codes “RPH” and “IUFD” were searched. The diagnosis had to be clearly established in the medical records, with IUFD confirmed by the absence of cardiac activity on ultrasound. Exclusion criteria included cases of fetal death occurring before 22 weeks of amenorrhea, in accordance with the World Health Organization’s definition of IUFD, or a maternal age less than 18 years.

Data were extracted from the patients’ paper records. A non-opposition search was conducted by individual letter for patients who had not been informed about the use of their health data prior to delivery.

We collected descriptive data about the patients, as well as their medical history and information regarding the current pregnancy.

Factors that could influence the speed of delivery, such as a fast artificial rupture of membranes, the mode of labor induction, and multiparity, were noted. An artificial rupture of membranes was considered rapid if performed within 90 min of diagnosis.

The presence of a complication was defined based on a composite criterion consisting of:

• Transfusion of blood products,
• Severe anemia (Hemoglobin ≤ 9 g/dL),
• Intensive care unit stay,
• Coagulopathy.

Bleeding was classified as “severe” (≥1000 ml) or “non-severe” (<1000 ml), as reliable quantification of bleeding less than 500 ml was not always possible. Coagulation disorders were stratified by severity groups according to the CEMIR classification (15).

Comparisons of qualitative data were performed using the Chi^2^ test, or Fisher’s exact test for small sample sizes. Quantitative variables were compared using the Student’s *T*-test or the Wilcoxon test for small sample sizes. A significance threshold of 5% was used.

Data processing was carried out in accordance with the MR-004 methodology, which does not involve human subjects, as proposed by the French Commission on Informatics and Liberty. This study was approved by the Institutional Review Board of the University Hospital Center of Fort-de-France.

## 3 Results

During the study period, 42,184 births were recorded. Among them, 28 records had coding compatible with the study criteria. One record was excluded due to an event occurring before 22 weeks of amenorrhea. The prevalence of RPH complicated by IUFD was 6.4 per 10,000 births.

The patients had a mean age of 32.2 years and were, on average, at 31 weeks of gestation. The mean BMI was 24.7 kg/m^2^. One-third of the patients (33%) were primiparous. Three patients (13%) had a history of CD, including one patient with a history of two CDs. Two patients smoked and one patient was known and treated for substance abuse (crack).

A trial of VD was initiated in 26 out of 27 patients (96%), and successful VD occurred in 23 patients (85%). Among these 23 patients, 11 (48%) had coagulopathy at admission. As shown in Table 1, three patients (11%) required a CD during labor due to insufficient progress of labor considering the severity of coagulopathy. Only one cesarean section was performed without a trial of labor. This patient had very severe coagulopathy at admission, with thrombocytopenia of 28,000/ μl and a Bishop score of 1.

**TABLE 1:** Details of the cesarean delivery sub-group.

	A	B	C	D
Anesthesia	General	General	Epidural	General
Trial of labor	No	Yes	Yes	Yes
Suspected indication	Previous CD Coagulopathy	Coagulopathy Stagnation	Bleeding Stagnation	Breech Stagnation
Bishop	1	5	4	6
Bleeding	Yes	Yes	Yes	Yes
Labor Length (hours)	/	3.2	3.75	3.3
PPH (ml)	Yes (1500)	Yes (1800)	No (<500 ml)	No (<500 ml)
Severe anemia	Yes	Yes	Yes	Yes
Transfusion	5RCC 3FFP 2PT	4 RCC 8FFP	3 RCC 2FFP	No
Coagulopathy before expulsion	Yes	Yes	No	No
Coagulopathy after expulsion	Yes	Yes	Yes	No
ICU	Yes (1 day)	No	No	No
Length of stay	6 days	4 days	5 days	5 days

RCC, Red Cell Concentrate; FFP, Fresh Frozen Plasma; PT, Platelet Transfusion.

The ongoing pregnancy was complicated by preeclampsia in 44.4% of cases. Coagulation disorders were present at admission in 44.4% of cases, with severity classified as severe or very severe in 18.5% of cases.

Most patients (51.8%) received oxytocin alone for induction. A rapid artificial rupture of membranes was performed in 44.4% of patients. The average duration of labor was 12 h and 9 min, with a median of 6 h and 3 min. The duration of labor was significantly shorter in the CD subgroup, with a mean duration of 3.4 h ± 0.27 compared to 13.0 h ± 17.5 in the VD subgroup (*p* = 0.01). The average length of hospitalization was significantly longer in the CD group, with 5 ± 0.8 days compared to 3.1 ± 1.4 days in the VD group (*p* = 0.01).

Post-partum hemorrhage (PPH) was reported in 25.9% of cases. No patient required surgical management or radiological embolization.

A composite complication criterion was observed in two-thirds of cases. All patients who underwent CD had a complication included in the composite criterion (*p* = 0.12).

CD was responsible for severe hemorrhage in 50% of cases, compared to 21.7% for vaginal deliveries, although this difference was not statistically significant (*p* = 0.23).

The distribution of complications included in the composite criterion is presented in Table 2. Data comparing the groups of patients with and without complications are reported in Table 3.

**TABLE 2:** Results based on mode of delivery.

	VD (*N* = 23)	CD (*N* = 4)	*P*
Labor duration (hours; mean ± SD)	13.0 ± 17.5	3.4 ± 0.27	0.01
PPH > 1000 ml	5 (21.7%)	2 (50%)	0.23
Complication (*N*; %)	14 (60.1%)	4 (100%)	0.12
Transfusion (*N*; %)	8 (34.7%)	3 (75%)	0.33
ICU stay (*N*; %)	4 (17.4%)	1 (25%)	0.72
Hospitalization (days; mean ± SD)	3.1 ± 1.4	5 ± 0.8	0.01

**TABLE 3:** Results of the group with and without complication.

		Complications (*N* = 16)	No complication (*N* = 11)	*P*
**Demography**				
Age (years; mean ± SD)		32.7 ± 6.0	31.0 ± 8.34	0.53
BMI (Kg/m2, mean ± SD)		23.7 ± 3.6	26.8 ± 8.7	0.18
Parity (N; mean ± SD)		1.0 ± 0.9	1.1 ± 1.6	0.36
Gestational age (weeks, mean ± SD)		32.6 ± 4.6	30.12 ± 4.5	0.30
History of VD (*N*, %)		8 (50%)	5 (45%)	0.42
History of CD (*N*, %)		3(18.7%)	1(9.1%)	0.62
**Admission**				
Preeclampsia (*N*, %)		8 (50%)	4 (36.3%)	0.38
Bishop > 6 (*N*, %)		5 (31.2%)	3 (27.2%)	0.16
Bleeding		15 (93.7%)	6 (54.5%)	0.03
Coagulopathy	Moderate	7 (43.7%)	0	/
	Severe	2 (12.5%)	0	/
	Very severe	3 (18.7%)	0	/
**Labor**				
Duration (hours; mean ± SD)		17.9 ± 22.1	9.2 ± 12.5	0.20
Fast Rupture of membranes (*N*; %)		1 (4.5%)	8 (38.1%)	0.07
Oxytocin induction (*N*, %)		8 (50%)	4 (36.3%)	0.57
Cesarean delivery (*N*, %)		4 (25%)	0	0.12
**Anesthesia**				
General (*N*, %)		3 (18.7%)	0	0.25
Epidural (*N*, %)		7 (43.7%)	9 (82%)	0.11
Others (*N*, %)		6 (37.5%)	2 (18.1%)	0.40
**Hemorrhage**				
Fibrinogen (mean; g/L ± SD)		2.58 ± 1.34	3.87 ± 1.1	0.01
Fibrinogen IV (*N*, %)		4 (25%)	0	0.12
Tranexamic acid IV (*N*, %)		4 (25%)	0	0.12
Surgery or embolization		0	0	/
**Stay**				
Hospitalization (mean, hours ± SD)		3.6 ± 1.6	2.8 ± 0.8	0.11

The duration of labor was not significantly correlated with the occurrence of complications (*p* = 0.20). A low fibrinogen level at admission was associated with increased complications (*p* = 0.01) and severe PPH (*p* = 0.01). This association was also observed in the VD subgroup (*p* = 0.01). The presence of bleeding at admission was correlated with an increased risk of complications (*p* = 0.03). Other data studied were not significantly associated with the occurrence of complications or severe PPH.

A history of VD, oxytocin-induced labor, and rapid artificial rupture of membranes were associated with faster delivery (*p* = 0.03). Only oxytocin induction was also associated with a decrease in the risk of severe PPH (*p* = 0.01). None of the patients induced with oxytocin presented severe PPH.

## 4 Discussion

Our study presents the results of a series of 27 cases of RPH complicated by IUFD over a period of approximately 20 years. The incidence of 6.4 per 10,000 births observed in our study is similar to that reported in the literature. The national Japanese study by Wada et al. indicates an incidence of 7.6 per 10,000 births, while a study from France reports an incidence of 3.33 per 10,000 births (3, 16).

The median duration of labor, approximately 6 h, is consistent with another Japanese series (17).

The 44% preeclampsia rate in our study is more than 10 times higher than that of the general population underlining it as a prime risk factor. Only one patient declared consuming crack and two patients declared smoking, no hypotheses can be drawn from these results.

Our initial hypothesis, based on pathophysiology, was that there would be a link between the duration of labor and the occurrence of complications, and that there might be a threshold beyond which the rate of complications would increase. However, we did not find a significant association between the duration of labor and the occurrence of complications or severe PPH.

One possible explanation could be that complications occur early and not progressively. The results from Inoue et al.’s series support this idea, showing an association between prolonged labor and a lower volume of bleeding, which was significant in their study (17). The authors suggest that the longer duration of labor allows for correction of coagulation disorders prior to delivery. In our series, three patients received fresh frozen plasma before expulsion; however, this does not provide sufficient evidence to support the hypothesis proposed by the Japanese authors.

Another hypothesis is that in cases of rapid labor, uterine contractions might be stronger or qualitatively different, potentially facilitating the passage of tissue thromboplastin or amniotic fluid into the bloodstream, thereby exacerbating the coagulopathy.

A history of VD and a rapid artificial rupture of membranes were associated with shorter delivery times but did not affect the occurrence of complications or the risk of severe PPH. It may not be the duration of labor, but rather its quality and individual susceptibility that play a role. The method of induction might also influence the passage of tissue thromboplastin into the bloodstream, as well as the possible uterine atony component that could exacerbate bleeding (18, 19). Notably, the group induced with oxytocin had significantly fewer cases of severe PPH, experienced faster labor, but did not show a notable reduction in the incidence of complications. Our data do not provide further support for this hypothesis.

With 85% of VD and 15% of CD, our results are comparable to those of Inoue et al. (17). VD is possible even in cases of severe coagulopathy at admission with acceptable morbidity, as reported in the literature (16, 17, 20). Indeed, among the patients who delivered vaginally in our series, 50% had coagulopathy. The rate of severe PPH was 21.7%, with 34% of patients requiring a transfusion, 17% admitted to the ICU, and a mean length of hospital stay of 3.1 days.

All CD performed during labor were due to insufficient progression, considering the coagulopathy. The duration of labor was shorter in these cases, probably because they were more severe at admission, prompting the decision for CD. The risks associated with coagulopathy may be overestimated, and more aggressive management, including transfusions before expulsion, could potentially delay and possibly avoid CD. In fact, the modest increase of 2 days in average hospital stay between CD and VD supports this idea, as this observed difference mirrors data from the 2019 French report on delivery statistics (21). Furthermore, there is significant variation in the incidence of CD for this complication, ranging from 8.2% in Belgium to 55% in France (16, 22). The case of Japan is interesting, as the CD rate decreased from 87.5% in 2002 to 66.7% in 2008, and then to 53% between January 2013 and December 2019 (3, 17). The indication seems to depend on local practices and technical capabilities.

Plasma fibrinogen is a known prognostic factor, especially in severe PPH (10, 11). The fibrinogen level at admission was significantly lower in the group with complications and severe bleeding. The study by Atallah et al. shows a similar association, notably with a fibrinogen level lower than 1.9 g/L, aligning with the average of 1.30 g/L observed in our data (16). However, this association was not found in the study of Inoue et al. (17). Several reports have suggested correcting coagulation disorders before delivery, but the low utilization of these measures in our series does not allow us to confirm their utility.

Our team now believes that there may be a genetic or acquired predisposition that promotes the occurrence of complications. Some patients experience complications, while others do not. In this context, the duration of labor may not be the central factor.

The recent French recommendations do not provide a specific management protocol for this context (12). We believe that a standardized management protocol could help to unify the VD rate in these cases by rationalizing the use of CD while avoiding an increase in morbidity.

A larger-scale study aimed at confirming our hypothesis that correcting coagulation disorders during antepartum care could reduce the occurrence of complications, particularly bleeding, is warranted.

## 5 Limitations

Our study has several limitations. Due to the low prevalence of this condition, we were only able to conduct a retrospective analysis of a limited number of records. The small sample size reduces the power of our results and prevented us from comparing the two delivery modes in a statistically significant manner. Moreover, the retrospective nature of the analysis led to substantial data loss, especially regarding essential management data such as bleeding, which was not always well documented. As a result, some patients were not classified as having PPH despite receiving two units of red blood cells. In four cases, the information was poorly recorded, which may have introduced bias in patient classification and thus skewed some of the results.

## 6 Conclusion

VD was successful in 85% of patients, with a complication rate of 60%, including 21% severe PPH. These results suggest that VD is feasible without a significant increase in risk for patients. Coagulopathy was present in 52% of patients, which suggests that it is not a formal contraindication to VD.

Additionally, the duration of labor does not appear to negatively influence maternal morbidity and mortality. Plasma fibrinogen levels at admission are an important prognostic factor for the risk of complications and severe hemorrhage.

The importance of proactive and aggressive management of coagulopathies may contribute to a further reduction in complications and an increase in VD.

Due to our low CD rate and the small size of our sample, we were unable to definitively recommend one mode of delivery over another.

## Data Availability

The raw data supporting the conclusions of this article will be made available by the authors, without undue reservation.
